# Computing muscle mechanical state variables from combined proprioceptive sensory feedback

**DOI:** 10.1113/EP092351

**Published:** 2025-06-23

**Authors:** Jacob D. Stephens, Lena H. Ting, Timothy C. Cope

**Affiliations:** ^1^ Coulter Department of Biomedical Engineering Emory University and Georgia Institute of Technology Atlanta Georgia USA; ^2^ Department of Rehabilitation Medicine Emory University Atlanta Georgia USA; ^3^ School of Biological Sciences Georgia Institute of Technology Atlanta Georgia USA

**Keywords:** computational modelling, Golgi tendon organ, muscle spindle, proprioception

## Abstract

Proprioceptive sensory feedback is crucial for the control of movement. In many ways, sensorimotor control loops in the neuromuscular system act as state feedback controllers. These controllers combine input commands and sensory feedback regarding the mechanical state of the muscle, joint or limb to modulate the mechanical output of the muscles. To understand how these control circuits function, it is necessary to understand fully the mechanical state variables that are signalled by proprioceptive sensory (propriosensory) afferents. Using new computational approaches, we demonstrate how combinations of group Ia and II muscle spindle afferent feedback can allow for tuned responses to force and the rate of force (or length and velocity) and how combinations of muscle spindle and Golgi tendon organ feedback can parse external and internal (self‐generated) force. These models suggest that muscle spindle feedback might be used to monitor and control muscle forces in addition to length and velocity and, when combined with tendon organ feedback, can distinguish self‐generated from externally imposed forces. Given that these models combine feedback from different sensory afferent types, they emphasize the utility of analysing muscle propriosensors as an integrated population, rather than independently, to gain a better understanding of propriosensory–motor control. Furthermore, these models propose a framework that links neural connectivity in the spinal cord with neuromechanical control. Although considerable work has been done on propriosensory–motor pathways in the CNS, our aim is to build upon this work by emphasizing the mechanical context.

## Abstract (highlights)

- **What is the central question of this study?**
  How can combinations of feedback from multiple propriosensor types signal muscle mechanical state variables for control?
- **What is the main finding and its importance?**
  Combined feedback from muscle spindle afferents can parse static and dynamic components of muscle stretch, and combined feedback from muscle spindle and tendon organ afferents can dissociate external forces from forces generated internally by contraction. We propose these mechanical state variables as potential control variables in sensorimotor circuits.

## 1 INTRODUCTION

Sensory feedback from muscle proprioceptive sensors [propriosensors; the group Ia and II muscle spindle (MS) afferents and group Ib Golgi tendon organ (GTO) afferents] is crucial for the control of movement. In extreme cases, loss of this feedback leaves seemingly automatic tasks, such as locomotion, possible only through considerable conscious effort (Rothwell et al., 1982). Many motor disorders are associated with dysregulation of the propriosensory–motor loop, but whether these arise from sensory, central or motor dysfunction can be difficult to ascertain. For example, peripheral axotomy has been shown to result in motor impairment despite the regeneration of sensory and motor axons (Abelew et al., 2000; Maas et al., 2007; Pantall et al., 2016; Sabatier et al., 2011), probably owing, in part, to synaptic changes in the spinal cord. However, the link between the disruption of this pathway, changes in spinal connectivity and the resulting change in motor control remains unclear.

**FIGURE 1:**
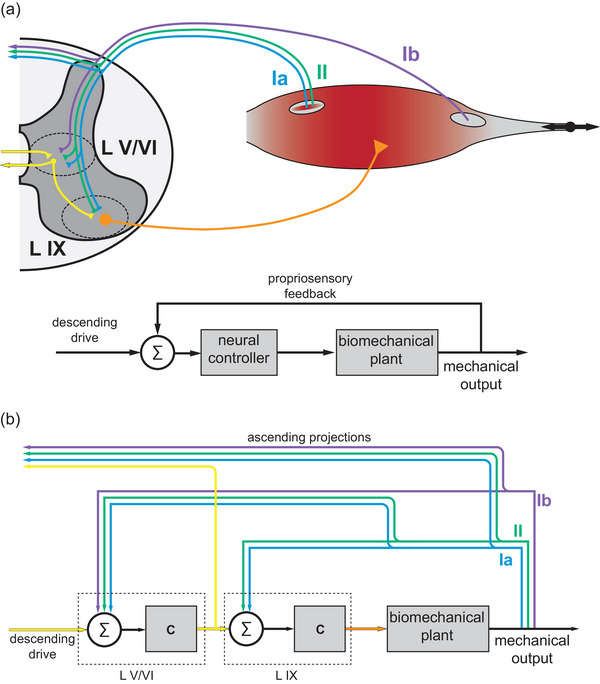
Schematic diagram of physiological propriosensory–motor circuits (adapted from Vincent et al., 2017) in the spinal cord and corresponding circuit model diagrams. (a) Group Ia (blue) and group II (green) muscle spindle afferents project to motor neurons (orange) in lamina IX and project to ascending circuits and interneurons in laminae V and VI (or the deep dorsal horn) alongside group Ib afferents (purple) from Golgi tendon organs. Interneuron circuits receive ascending input from other areas (yellow) and relay signals (yellow) to ascending areas and to motor neurons. This can generally be represented by the state feedback control model, where feedback is integrated with descending commands to modulate command signals to the biomechanical plant, in this case the muscle. As a note, this model is a general representation of closed‐loop control and is not specific to the spinal circuits in question, and can represent control schemes at various levels of the CNS. (b) Circuit model diagram of a homonymous propriosensory–motor loop within the spinal cord. Depicted are the parallel loops of muscle spindle feedback through lamina IX and muscle spindle and Golgi tendon organ feedback through the deep dorsal horn. The motor neurons in lamina IX are depicted as a controller and summing junction for muscle spindle propriosensory feedback and motor signals from interneuron circuits. The interneurons are modelled as a controller and summing junction for propriosensory feedback from muscle spindles and tendon organs and descending drive from elsewhere in the CNS. Abbreviations: c, controller; Σ, summing junction.

Computational approaches offer some promise in analysing these systems. One useful model of propriomotor integration is a state feedback control system (Scott, 2012). The benefit of this model is in mapping an input sensory stimulus to a motor output (Figure 1a), which is readily available experimentally via measures such as EMG. Such models can better link physiology and behavior by establishing relationships between mechanics, sensory encoding and motor responses. However, sensory feedback signals are not readily available experimentally, and models of mechanical information signalled by propriosensory afferents are incomplete. Although generally understood as length sensors, muscle spindles contain intrafusal muscle fibres that lend them force‐encoding characteristics, which can decouple their signals from length and velocity (Blum et al., 2017, 2019, 2020; Nichols & Cope, 2004; Poppele et al., 1979; Proske et al., 2014). Whether muscle spindles signal force‐ or length‐related information would also change how this information is integrated with muscle force feedback from GTOs.

Some of the questions regarding the mechanical state variables signalled by propriosensors could be addressed better by considering these sensors as parts of a population. For example, motor neurons receive direct input from both group Ia and II MS afferents. Although the contributions of the group Ia afferent to this circuit (the monosynaptic stretch reflex) are well known, there is also evidence of group II afferent contributions that warrant attention (Banks et al., 2021; Kanda & Rymer, 1977), and the functional role of the monosynaptic stretch reflex might be understood better by considering this combined feedback. Additionally, MS feedback alone cannot distinguish between a passive stretch and an eccentric contraction, even though these are different mechanical states of the muscle. Differentiating them requires combinination of MS and GTO feedback. Physiological evidence that the signals are combined is found in the convergence of MS and GTO input to interneuron populations in the spinal cord, such as interneurons in the deep dorsal horn and Clarke's column (Bosco & Poppele, 2001; Jankowska & Edgley, 2010). Computational studies have also shown that combinations of signals from multiple propriosensor types predict joint velocities better than single propriosensors (Dimitriou & Edin, 2008) and can explain features of motor output better (Nagamori et al., 2018). Additionally, studies of the vestibulomotor system show that exafferent (stimulus‐driven) and reafferent (self‐generated) sensory stimuli are differentiated by combining multiple sensory inputs (Cullen, 2012), suggesting that relevant control signals might be computed from combined feedback rather than directly encoded by sensors.

In this article, we demonstrate how the signals from propriosensory afferents can be combined to extract behaviourally relevant mechanical state variables. Using simple computational approaches together with in situ sensory afferent recordings, we demonstrate that combined group Ia and II MS afferent feedback can differentiate static and dynamic components of a stretch stimulus. In addition, we propose that the combination of MS and GTO feedback enables the dissociation of self‐generated and externally‐imposed forces during eccentric contraction.

## 2 MATERIALS AND METHODS

### 2.1 Ethical approval

All animals were cared for under Georgia Institute of Technology Institutional Animal Care and Use Committee animal care and use procedures (protocol number A100142). Animals were housed in clean cages and given food and water ad libitum. Animals were adult female Wistar rats sourced from Charles River. All animals were anaesthetized with 5% isoflurane until the toe pinch reflex was absent and killed via exsanguination. Data from a single animal are analysed here, with the inclusion criterion being the availability of data from all three proprioceptive muscle afferent types for each stretch repetition. The animal in question had a body weight of 277 g.

### 2.2 Data collection

Terminal surgical procedures were conducted as described in previous experiments reported from this laboratory (Vincent et al., 2017). The triceps surae muscle–tendon unit was isolated and fixed to a servo‐controlled motor (Aurora 305C‐LR, Aurora Scientific, Aurora, ON, Canada) via kevlar thread. The change in length of the muscle–tendon unit (MTU) and the resulting forces were recorded from the motor. Sonomicrometry crystals (Sonometrics, London, ON, Canada) were implanted in the medial gastrocnemius to record the internal change in length of the muscle. Afferent recordings were taken intracellularly via glass micropipettes. Afferents were classified as Ia, II or Ib based on responses to twitch contractions and entrainment of muscle vibration at ≥100 Hz. Muscle stretches consisted of 3 mm ramp–hold–release with a 1 s hold. Ramp velocities were set at 15, 17.1, 20, 24 and 30 mm/s, and three stretches were collected at each velocity. The example group Ia, group II and group Ib afferent recordings shown here were selected based on their consistent responses to stretch at each velocity and availability of the full stretch protocol (three stretches at each velocity, *n* = 15 stretches per cell and *n* = 3 cells).

### 2.3 Data processing

Data were collected using CED Spike2 software (Cambridge Electronic Design, Cambridge, UK) and exported to MATLAB (Mathworks Inc., Natick, MA, USA) for analysis and modelling. Data were recorded at 17.8 kHz, downsampled to 1.78 kHz, then low‐pass filtered using a fourth‐order Butterworth filter with a 100 Hz cut‐off. Derivatives were computed using a second‐order Savitsky–Golay filter with a window width of 51 samples (∼29 ms).

To combine instantaneous firing rates linearly, the instantaneous firing rates were linearly interpolated to a 1 kHz time vector. To ensure that signals were time aligned, signals of each afferent type were matched by velocity. The time vector started at the first recorded spike after stretch onset, typically the initial burst from the group Ia. The rate vectors for the groups II and Ib were set to be zero initially, unless there was background firing, in which case the rate vector was initialized at the last instantaneous rate recorded before stretch.

### 2.4 Computational modelling

**FIGURE 2:**
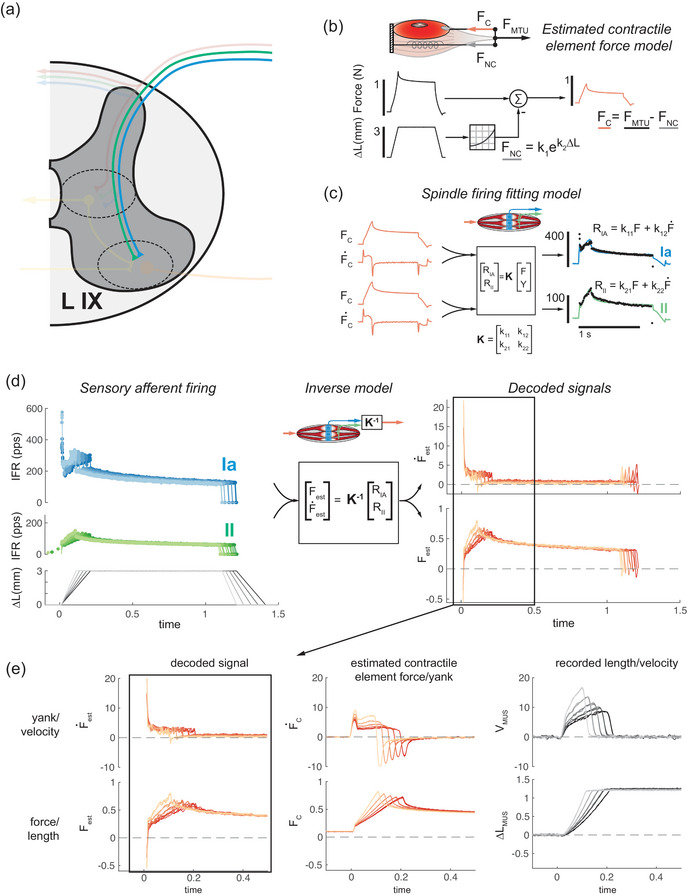
De‐mixing mechanical state variables from combined muscle spindle feedback. (a) Reproduction of Figure 1a with the specific monosynaptic circuit in question identified, as this circuit combines feedback from the group Ia and II MS afferents. (b) As described by Blum et al. (2019), the contribution of non‐contractile tissue to the total force of the muscle is estimated using an exponential force–length model and subtracted from the total force of the MTU. (c) The remaining estimated force on contractile tissues of the muscle ($F_{C}$) and its derivative, termed yank ($\dot{F_{C}}$), are fitted to the firing rates of group Ia and II afferents using the method described by Blum et al. (2017). (d) Using these model coefficients, an inverse matrix can be obtained to map measured spike rates of muscle spindle afferents into estimates of the driving mechanical variables (described in detail in Section 2). (e) Comparison of sensory estimates of mechanical signals shows similarities to the $F_{C}$ and $\dot{F_{C}}$ obtained during stretch at various velocities. Other qualitative similarities can be observed between the sensory estimates of $F_{C}$ and $\dot{F_{C}}$ and the length change and velocity of the muscle recorded via sonomicrometry, namely in the velocity‐scaling of the sensory estimate force and consistent value during the hold period that matches the fascicle length. Abbreviations: MS, muscle spindle; MTU, muscle–tendon unit.

**FIGURE 3:**
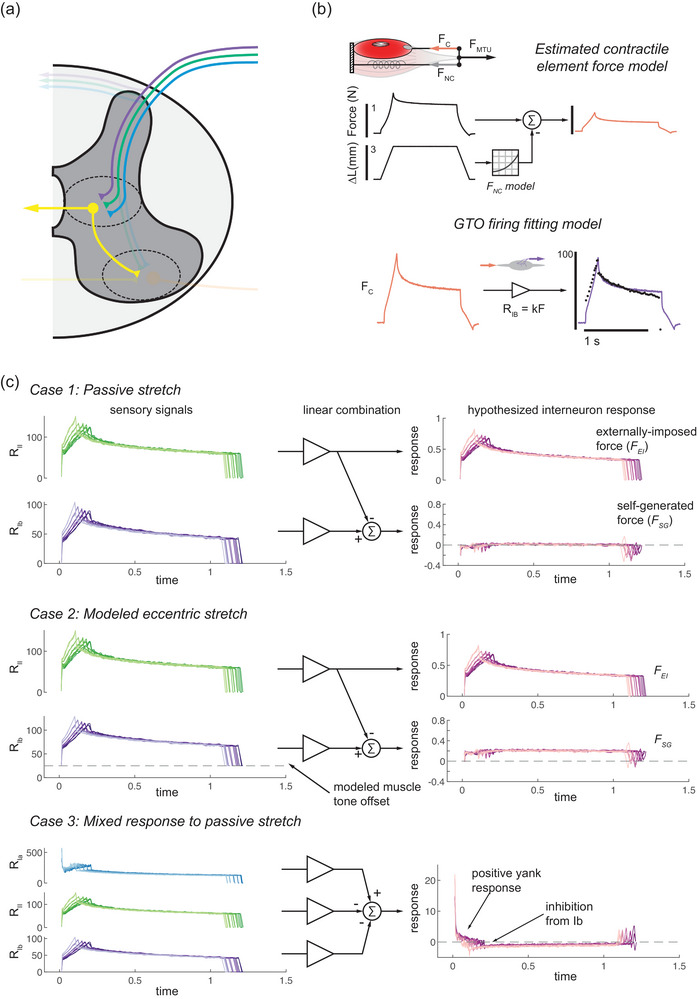
De‐mixing mechanical state variables from combined muscle spindle and Golgi tendon organ feedback. (a) Reproduction of Figure 1a with the interneuronal feedback circuit in question highlighted, as it combines the feedback from all three types of propriosensory afferents. (b) The same process of subtracting the estimated contribution of non‐contractile muscle tissues from total MTU force is repeated. (c) The estimated contractile element force ($F_{C}$) is fitted to the recorded firing rate of a group Ib Golgi tendon organ afferent to obtain an estimate of the group Ib sensitivity to force, using the same process as in Figure 2. (d) Using the force sensitivities of group II and Ib afferents, the firing rates can be combined linearly to cancel out the forces signalled by the group II and Ib afferents during passive stretch. During a modelled eccentric contraction, these forces no longer cancel out. The difference between them corresponds to the self‐generated force in the muscle ($F_{\mathrm{SG}}$), with the group II afferent signalling the externally imposed force ($F_{\mathrm{EI}}$) from the stretch. By combining these signals with those of a group Ia afferent, neurons can respond to the dynamics of self‐generated and externally imposed forces. Abbreviation: MTU, muscle–tendon unit.

Two primary modelling procedures were used in this paper. The first involved fitting mechanical data to afferent instantaneous firing rates in the manner described by Blum et al. (2017, 2019). In short, contributions of extramysial tissue to the total force of the MTU are estimated using an exponential force–length model based on MTU length and subtracted from the total MTU force (Figures 2b and 3b). Given that this model estimates the force on contractile muscle tissue, the model parameters were optimized to fit the firing rate of the Golgi tendon organ afferent (Figure 3b), and the only parameter changed to fit the group Ia and II data was the resting length such that the force–length curves were approximately matched. Contractile element force and yank estimates were optimized to fit recorded instantaneous firing rates, producing a set of optimal model gains (Figures 2c and 3c). These analyses were based on data from a single animal owing to the availability of full sets of ramp–hold–release stretches in at least one of each afferent type.

The second method used the gains obtained previously to infer the encoded state variables. The force and yank coefficients from the 15 mm/s stretches were averaged to create the *K* matrix (Figure 2c), which for the analysis in Figure 2 is:

(1) $$K=\left[\begin{array}{cc} 337.8 & 33.6\\ 182.8 & 4.9 \end{array}\right]$$

Then, instantaneous firing rates were multiplied by the inverse of the *K* matrix to de‐mix the signals and obtain estimates of force and yank. For the analysis in Figure 3, the scaling coefficients used

(2) $$k_{F,\mathrm{II}}=182.8\frac{\mathrm{spikes}}{N}\times s$$

(3) $$k_{F,\mathrm{Ib}}=128.8\frac{\mathrm{spikes}}{N}\times s$$

which are the estimated force gains of the group Ib and II afferent in question, and mechanical state variables were estimated by dividing by force gains.

## 3 RESULTS

### 3.1 Assertion 1: Stretch dynamics can be parsed from muscle spindle feedback

The simplest propriomotor loop is the monosynaptic stretch reflex, which arises from connections made by MS afferents to α motor neurons. In the absence of other inputs, the response of the motor neuron to a stimulus at the muscle depends on the combined input of the group Ia and II MS afferents. This suggests that the combination of group Ia and II MS afferents signals relevant information on the mechanical state of the muscle. The combination of this information is also necessary through the lens of feedback control. Given that group Ia afferents signal both the rate and the degree of muscle stretch, the motor neuron would have no way to discern how much the muscle was stretched versus how quickly it was being stretched without simultaneous feedback from a second sensor. By combining group Ia and II feedback, the motor neuron can parse the degree of stretch from the rate of stretch, because the group II afferent has a different sensitivity to the static and dynamic components of stretch in comparison to the group Ia (Cooper, 1961).

Thus, the relationship or ratio between the instantaneous firing rates of group Ia and II afferents is what encodes the static and dynamic components of stretch. This scheme can be represented mathematically by Equations (4) and (5):

(4) $$R_{\mathrm{Ia}}=k_{11}X+k_{12}\dot{X}$$

(5) $$R_{\mathrm{II}}=k_{21}\;X+k_{22}\dot{X}$$

where R is the response for an afferent (as an instantaneous firing rate), X is the degree of stretch, $\dot{X}$ is the rate of stretch, and the k values indicate sensitivities to the static and dynamic components of stretch. Given that the group Ia and II afferents share a common origin (Banks et al., 2021) and encode the same stretch, Equations (4) and (5) can be combined in the linear matrix equation:

(6) $$\left[\begin{array}{c} R_{\mathrm{Ia}}\\ R_{\mathrm{II}} \end{array}\right]=K\left[\begin{array}{c} X\\ \dot{X} \end{array}\right]$$

where R is a vector that now represents a sensory response along two separate pathways (groups Ia and II), and K is a matrix of coefficients mapping the static (X) and dynamic ($\dot{X}$) components of a stretch stimulus to the response R. Then, estimates of the individual state variables can be obtained by inverting Equation (6) into

(7) $$\left[\begin{array}{c} X_{\mathrm{est}}\\ {\dot{X}}_{\mathrm{est}} \end{array}\right]=K^{-1}\;\left[\begin{array}{c} R_{\mathrm{Ia}}\\ R_{\mathrm{II}} \end{array}\right]$$

where $X_{\mathrm{est}}$ and ${\dot{X}}_{\mathrm{est}}$ are sensory estimates of the input stimulus based on the response of group Ia and II afferents.

The results of implementing this process are shown in Figure 2. The models proposed by Blum et al. (2017, 2019) serve as the basis, using the estimated force on the contractile apparatus of the muscle (Figure 2a) during passive stretch as a proxy for the force on the MS, thus driving MS afferent firing (Figure 2b). Thus force (F) and yank ($\dot{F}$) can be substituted into Equation (4) and fitted to firing rates to populate the K matrix of coefficients (Equation 6). After training the model (details given in Section 2), the process was implemented in reverse (Equation 7), with instantaneous firing rates from group Ia and II afferents measured at different stretch velocities to obtain an estimate of the stimulus (Figure 2c). The decoded force has a similar profile and velocity scaling to the contractile element force ($F_{C}$). The decoded yank has the same profile as the contractile element yank (${\dot{F}}_{C}$ but does not show the same velocity scaling as the contractile element yank (Figure 2d, e). However, the velocity scaling of the decoded variables bears qualitative similarities to recorded length and velocity (Figure 2e), suggesting that length and velocity information could also potentially be extracted from this feedback.

This model demonstrates that these signals can be combined to extract and compute the static (force and/or length) and dynamic (yank and/or velocity) components of stretch. Given that these components are separable from the incoming neural signals, this suggests that propriomotor control loops can be tuned to respond to these components individually. In other words, the reflex response of the motor neuron can be tuned to force and yank (or length and velocity) individually by modulating the relative inputs of the group Ia and II afferents.

### 3.2 Assertion 2: Muscle kinetics can be computed from spindle and GTO feedback

Although combined feedback from group Ia and II MS afferents can signal stretch dynamics, GTO feedback is required to compute muscle‐level mechanical state variables. Combined MS and GTO afferent feedback has been hypothesized to signal MTU kinematics by estimating muscle length from MS afferents and tendon length from the force signalled by GTO afferents (Kistemaker et al., 2013) and to maintain limb stiffness (Nichols & Houk, 1976). Other work has shown that combined MS and GTO feedback offers a better estimate of joint kinematics during human reaching tasks (Dimitriou & Edin, 2008) and in simulations of compliant robotic limbs (Hagen et al., 2021), and has been hypothesized to play a role in effort perception (Monjo & Allen, 2023). However, the force‐encoding characteristics of MS afferents during stretch might also allow for computations of force‐related mechanical state variables.

As mentioned earlier, differentiating whether a muscle is being passively stretched or eccentrically contracting would require feedback from both the MS and GTO. During stretch, the force of the muscle can be expressed as:

(8) $$F_{\mathrm{MTU}}=F_{C}+F_{\mathrm{NC}}$$

where $F_{\mathrm{MTU}}$ is the total force in the MTU, $F_{C}$ is the force on contractile muscle tissue, and $F_{\mathrm{NC}}$ is the force on non‐contractile extramysial tissue as is depicted in the model in Figure 2b. In an eccentric contraction, some force will be attributable to the contraction of the muscle and some to the imposed muscle stretch. Force on the contractile tissue ($F_{C}$) can be broken into self‐generated (SG) and externally‐imposed (EI) components:

(9) $$F_{C}=F_{\mathrm{SG}}+F_{\mathrm{EI}\;}$$

where $F_{\mathrm{SG}}$ is the force actively generated by the muscle owing to contraction and $F_{\mathrm{EI}}$ is the force imposed on the muscle by the external stretch or perturbation. Because MSs have force‐encoding characteristics during stretch, they can be thought to signal $F_{\mathrm{EI}}$, and GTOs signal $F_{C}$ as they respond during stretch and contraction. Thus, the relative difference in the MS and GTO signals represents the $F_{\mathrm{SG}}$ generated by muscle activation.

Figure 3 shows how signals from the group II and Ib afferents can be combined to compute these variables. As in Figure 2, estimated non‐contractile force contributions are subtracted from the total MTU force, and the contractile element force is fitted to the group Ib firing rate such that:

(10) $$R_{\mathrm{Ib}}=k_{F,\mathrm{Ib}}\;F_{C}$$

The group II can also be approximated by force such that:

(11) $$R_{\mathrm{II}}\approx k_{F,\mathrm{II}}F_{\mathrm{EI}}$$

These can be used to approximate the internal force by:

(12) $$F_{\mathrm{SG}}=F_{C}\;-\;F_{\mathrm{EI}}=\frac{1}{k_{F,\mathrm{Ib}}}\;R_{\mathrm{Ib}}-\frac{1}{k_{F,\mathrm{II}}}R_{\mathrm{II}}$$

During these passive stretches, there should be no self‐generated force component, and subtracting the $F_{\mathrm{EI}}$ estimated from the MS and $F_{C}$ from the GTO should result in no self‐generated force signal. This computation is shown in Figure 3. The firing rate of the group II afferent can be divided by $k_{F,\;\mathrm{II}}$ ($k_{F,\;\mathrm{II}}$ = 182.8 spikes/N s) to estimate $F_{\mathrm{EI}}$, and the firing rate of the group Ib afferent can be divided by $k_{F,\;\mathrm{Ib}}$ ($k_{F,\;\mathrm{Ib}}$ = 128.8 spikes/N s) to estimate $F_{C}$. Then, the estimate of $F_{\mathrm{EI}}$ can be subtracted from the estimate of $F_{C}$, and as predicted, these force signals largely cancel out. Next, modelling an eccentric contraction by adding an offset to the group Ib afferent firing rate results in a non‐zero estimate of $F_{\mathrm{SG}}$, suggesting the relative difference in these signals can indicate the force generated by the muscle from the external force exerted on the muscle.

Given that interneuron circuits also receive input from group Ia afferents, an example response is shown with input from all three afferent types (Ia, II and Ib). By including the Ia, the hypothesized neuron response now incorporates the dynamics of the stretch stimulus in addition to the internal and external mechanics. The hypothesized response in Figure 3c shows activation by the yank of the stretch and inhibition by group Ib signals, which could effectively be considered a ‘startle’ response with behaviourally relevant implications, because the neuron would be activated preferentially by sudden external stimuli and inhibited by compensatory action. It also stands to reason that this mixed response could be varied for other neurons to respond to combinations of external stretch dynamics and self‐generated and external forces.

## 4 DISCUSSION

Overall, this work demonstrates how relevant mechanical state variables can be extracted or computed by simple combination; specifically, how static and dynamic components of stretch can be parsed by combined MS afferent feedback and how internal and external mechanics can be parsed by combined MS and GTO feedback. The aim of this work is to advance the understanding of the relationship between sensory physiology, limb mechanics and motor action. Lockhart and Ting (2007) showed that a loss of group I afferents in cats resulted in the inability to control the acceleration of the centre of mass during balance perturbations, demonstrating a link between a class of sensory afferents (in this case, group I) and alterations to the sensorimotor control scheme. Models such as those proposed here, based on the encoding of mechanical state variables by multiple classes of sensory afferents, can allow for exploration of the links between biomechanics, sensory and motor function. As mentioned previously, peripheral axotomy results in difficulty during downslope walking, owing, in part, to the reorganization of group Ia synapses on motor neurons and premotor interneurons (Abelew et al., 2000; Alvarez et al., 2020; Horstman et al., 2019; Sabatier et al., 2011). However, current models do not explain fully why motor control deficits are more pronounced in downslope walking, where eccentric contractions predominate or why the spared pathways fail to compensate. By linking physiology and limb mechanics, these deficits could be studied more accurately through the lens of loss of yank or velocity feedback in specific proprioceptive pathways to offer a more mechanistic explanation for the observed impairments.

We showed that combining group Ia and II MS afferent feedback can yield independent estimates of the static and dynamic components of stretch by simple linear combination. The estimated mechanical variables were similar to the original estimated contractile element force and yank and were also qualitatively similar to the recorded fascicle displacement and velocity. Although a similar model based on length and velocity would fail to account for the initial burst, the observed similarities warrant further exploration. To improve the fit between estimated force and yank and the afferent firing rates, contributions from extracellular matrix tissue within the muscle were subtracted as in prior work by Blum et al. (2019). Given that this subtraction also yielded more accurate fits to the firing rate of the group Ib GTO afferent (Figure 3b), this raises additional questions about force sensation in MTUs with dense extramysial tissue.

Although the models shown here treat MS afferents as force encoding, this does not discount their role in signalling length and velocity. The key distinction lies in what is being encoded and what is being sensed. Through their physical connections with intrafusal fibres, the deformation of the sensory terminals of MS depends on the forces in these intrafusal fibres (Bewick & Banks, 2015). Because muscle expresses viscoelastic properties, the forces encoded by MS afferents will resemble combinations of length and velocity. The findings of Proske et al. (2014) suggest that higher‐level neural processing extracts length information from MS feedback. However, the thixotropic properties of intrafusal muscle fibres introduce sensory errors when they do not behave like a viscoelastic system. Thus, whether MS feedback is best interpreted as representing muscle length–velocity or force–yank feedback should depend on the behavioural task and sensory systems being investigated. Models that can reproduce the thixotropic firing behaviours of MS afferents (Blum et al., 2020, Simha & Ting, 2023) offer a promising tool for investigation.

We also show that the combined input from group II and Ib afferents could parse self‐generated and externally imposed forces during stretch. However, it is important to reiterate that the eccentric contraction used in this study is hypothetical, because group II and Ib firing rates would probably look different in a true eccentric contraction. For this purpose, the eccentric contraction was modelled as a very small constant offset in the group Ib firing rate. Although the eccentric model used here might not perfectly represent groups II and Ib firing rates during an eccentric contraction, it does show that combined MS and GTO feedback can differentiate passive stretch and eccentric contraction, in addition to the self‐generated and externally imposed components. It has been suggested that the nervous system treats active and passive force differently (Savage et al., 2015), and the model shown here suggests that this process could arise from the encoding of muscle state by MS and GTO afferents. As a clarifying point, ‘self‐generated’ and ‘externally imposed’ are used with respect to the muscle and are not interchangeable with ‘exafferent’ and ‘reafferent’. If muscle stretch is elicited by the action of an antagonistic muscle, the stretch would be encoded as an externally imposed signal (with respect to the muscle), but the feedback would be reafferent because it arises from self‐motion. However, similar computations could be made across antagonistic muscles or muscle groups, potentially dissociating exafference and reafference. Likewise, MSs are classically defined as interoreceptors, signalling information regarding the internal state of the body, but they can also signal external perturbations applied to the muscle. In these contexts, it might be more fitting to consider MSs as exteroceptors, depending on the source of the stimulus.

The computations presented here are not definitive and can be adjusted across different levels and time scales. The γ motor neurons can increase the sensitivity of MS afferents to static and dynamic components of stretch individually (Matthews, 1962), in a span of milliseconds, and can be thought of as increasing responses to static and/or dynamic components of stretch depending on task demands. This modulation can vary reflex gain within the spinal cord over the course of minutes, and synaptic weighting can tune control strategies over considerably long time scales. This flexibility in control is also demonstrated by the distributed feedback in laminae V and VI, with different interneurons receiving different combinations of muscle propriosensory input. Figure 3c shows a hypothetical example of how feedback from all three muscle propriosensors might be integrated. These interneuron circuits might represent different functional pools that respond to variations of self‐generated and externally imposed forces, in addition to the dynamics of the external stimuli, with potential for individual modulation via descending inputs.

## 5 CONCLUSION

This work highlights the importance of viewing propriosensors as an integrated system that encodes the mechanical state of the muscle. This perspective can significantly advance both our models and the understanding of motor control. Although considerable work has explored the relationship between propriosensory deficits and motor control deficits, direct causal links are yet to be established. By uncovering how propriosensors collectively encode the mechanical state of the muscle, we can develop more accurate models to investigate how motor dysfunction arises from sensory deficits, central deficits and compensatory mechanisms.

## AUTHOR CONTRIBUTIONS

Jacob D. Stephens—data acquisition, conception and design, data analysis and interpretation, drafting and revising. Timothy C. Cope—data analysis and interpretation, conception and design, drafting and revising. Lena H. Ting—data analysis and interpretation, conception and design, drafting and revising. All authors have read and approved the final version of this manuscript and agree to be accountable for all aspects of the work in ensuring that questions related to the accuracy or integrity of any part of the work are appropriately investigated and resolved. All persons designated as authors qualify for authorship, and all those who qualify for authorship are listed.

## CONFLICT OF INTEREST

None declared.

## Data Availability

All data and code necessary to reproduce these results are available at github.com/stephensjake72/SpindleGrant/tree/main/JEP_2024.
